# Vertical marginal fit of advanced lithium disilicate crowns: an in- vitro study

**DOI:** 10.1186/s12903-026-07743-7

**Published:** 2026-03-03

**Authors:** Aisha Fouad Wafaie, Hanaa Hassan Zaghloul, Yara Sayed Attia, Tamer A. Hamza

**Affiliations:** 1 Department of Fixed Prosthodontics, Faculty of Oral and Dental Medicine, Misr International University, Cairo, Egypt; 2 Department of Fixed Prosthodontics, Faculty of Dentistry, Noura Bint Abdulrahman University, Riyadh, Saudi Arabia; 3 Department of Fixed Prosthodontics, Faculty of Oral and Dental Medicine, Misr International University, Cairo, Egypt; 4 Department of Fixed Prosthodontics, Faculty of Dentistry, Badr University in Cairo, Cairo, Egypt

**Keywords:** Vertical marginal fit, CEREC tessera, IPS emax CAD, Crowns

## Abstract

**Statement of the problem:**

The clinical performance of ceramic crowns is influenced by overall marginal adaptation. A recently introduced advanced lithium disilicate ceramic requires further evidence compared to conventional lithium disilicate in terms of vertical marginal fit.

**Purpose of the study:**

To assess the vertical marginal fit of crowns constructed from advanced and conventional lithium disilicate materials.

**Materials and methods:**

Ten (*n* = 10) ceramic crowns were constructed and randomly assigned to two groups: Group (T) with CEREC Tessera crowns (*n* = 5) and Group (E) with IPS e.max CAD crowns (*n* = 5). Vertical marginal fit was evaluated using a stereomicroscope at 10X magnification both before and after cementation, with twenty equidistant measurement points recorded for each crown. Each crown was cemented to its corresponding natural molar tooth using Totalcem resin cement. Mann-Whitney U test was used to compare the two material groups. Wilcoxon signed-rank test was used to compare between vertical marginal fit before and after cementation (*P* ≤ 0.05).

**Results:**

The overall vertical marginal fit between the two groups showed no significant difference, whether before or after cementation. However, both groups exhibited a statistically significant decrease in vertical marginal fit after cementation.

**Conclusion:**

Advanced lithium disilicate shows advantageous properties concerning mean marginal gap values exhibiting comparable performance to IPS e.max CAD.

**Clinical implications:**

CEREC Tessera demonstrated marginal fit values within clinically acceptable limits, supporting its reliability as a novel chairside material suitable for use in restorative dentistry.

## Introduction

Recent advancements in dental technology have led to the development of high-quality ceramic materials that are gaining popularity. These materials offer excellent esthetics, including attractive optical properties and natural tooth color, along with chromatic stability. They are also biocompatible and feature optimal mechanical characteristics, including high flexural strength, wear resistance, fracture toughness and low abrasiveness [1, 2].

The use of ceramic restorations has increased significantly due to advancements in computer-aided design and computer-aided manufacturing (CAD/CAM) technology. In modern dentistry, time efficiency and predictable treatment outcomes are greatly valued, and CAD/CAM technology excels in delivering these benefits. By minimizing processing times and offering superior flexural strength, this technology enhances workflow in dental practices. Additionally, it has enhanced the marginal fit, mechanical strength, and reliability of final restorations [1, 3].

The clinical longevity of ceramic restorations is influenced by multiple factors, including ceramic material composition, microstructural stability, fabrication method, marginal adaptation, cementation protocol and the patient’s oral environment. Among these factors, the choice of ceramic material plays a central role in determining long-term success. Lithium disilicate-based glass ceramics (LDCs) have become essential in restorative and esthetic dentistry due to their favorable combination of strength, translucency, and predictable clinical performance [4].

Lithium disilicate-based glass ceramics are used in various dental applications, including single-tooth restorations and full mouth rehabilitations. They are suitable for both anterior and posterior crowns, in addition to being used for fixed partial dentures up to the second premolars [5].

IPS e.max lithium disilicate-based glass ceramic (Ivoclar Vivadent, Schaan, Liechtenstein) is available in both ingot form for heat pressing and machinable blocks suitable for computer-aided design and computer-aided manufacturing. The IPS e.max CAD blocks are partially crystallized, which enhances their machinability. Available in various shades and translucency levels, its aesthetic appeal, high strength, and favorable processing features make it popular among dental professionals for diverse applications [4, 6–8].

Advanced lithium disilicate ceramic (CEREC Tessera) (Dentsply Sirona, Hanau-Wolfgang, Germany) has been recently launched in the dental market. This material features a special microstructure of lithium disilicate (Li2Si2O5) and virgilite crystals (Li0.5Al0.5Si2.5O6), within a zirconia-enhanced glass matrix. Additional virgilite crystals develop during firing at temperatures between 800 °C and 850 °C. CEREC Tessera is available as tooth-colored CAD/CAM blocks which require mandatory firing to achieve its final strength. A supplementary glaze firing at 760 °C for 4.5–12 min is necessary to enhance strength further. In comparison, IPS e.max CAD undergoes a different crystallization process [5, 9–14].

Vertical marginal fit plays a critical role in the long-term performance of ceramic restorations. McLean and von Fraunhofer [15]. reported that a clinically acceptable vertical marginal fit is less than 120 μm. Variations in marginal gaps will lead to luting agent exposure to the oral environment, potentially leading to its dissolution and marginal discoloration. The marginal precision of ceramic restorations is influenced by crystallization shrinkage, milling accuracy, scanner precision, and preparation design. Factors such as bur wear, material brittleness, and furnace calibration significantly affect edge quality and dimensional stability. Understanding these variables is essential for optimizing the precision and clinical performance of ceramic restorations [16–23].

Since CEREC Tessera and IPS e.max CAD differ in microstructure, crystallization shrinkage, and milling behavior, even small variations in marginal fit may translate into clinically significant differences in marginal stability and long-term success. Thus, assessing and comparing marginal fit is essential when choosing between these materials for durable, biologically compatible, and predictable restorative outcomes. Thus, the current study aimed to assess the vertical marginal fit of crowns constructed from advanced lithium disilicate (CEREC Tessera) compared to conventional lithium disilicate (IPS e.max CAD). The null hypothesis stated that there would be no significant difference in vertical marginal fit between the crowns of CEREC Tessera and IPS emax CAD.

## Materials and methods

Ten non-identified, sound, caries-free human molar teeth were obtained from the Teeth Bank of the Faculty of Oral and Dental Medicine, Misr International University, Egypt, following ethical approval from the Institutional Review Board (IRB) of the Faculty of Oral and Dental Medicine, Misr International University (Approval No. MIU-IRB-00010118; FWA No. 00022887). All procedures were conducted in accordance with the Declaration of Helsinki and the institutional ethical guidelines. The teeth were assessed to ensure they had approximately equal mesio-distal and inciso-cervical dimensions at the coronal portion. Each selected tooth was thoroughly examined with a magnifying lens. They were cleaned using an ultrasonic scaler to remove any deposits and soft tissues from the surface. The cleaned teeth were then stored in normal saline at room temperature in a sealed container [24].

Power analysis was centered on the marginal gap distance following cementation as the main outcome. As reported by Ahmed et al. [25] (2020), the mean and standard deviation values were 29.58 (0.64) for Group I and 21.33 (2.07) µm for Group II, resulting in an effect size (d) of 5.38. Because our study applied the same measurement variable, we used these published values as the empirical basis for the calculation. With an alpha (α) level set at 5% and a beta (β) level of 20% (power = 80%), the minimum sample size required was determined to be 3 specimens per group. To compensate for any potential overestimation of effect size, we increased the sample size to 5 specimens per group. Sample size calculation was performed using G*Power Version 3.1.9.2.

**Fig. 1:**
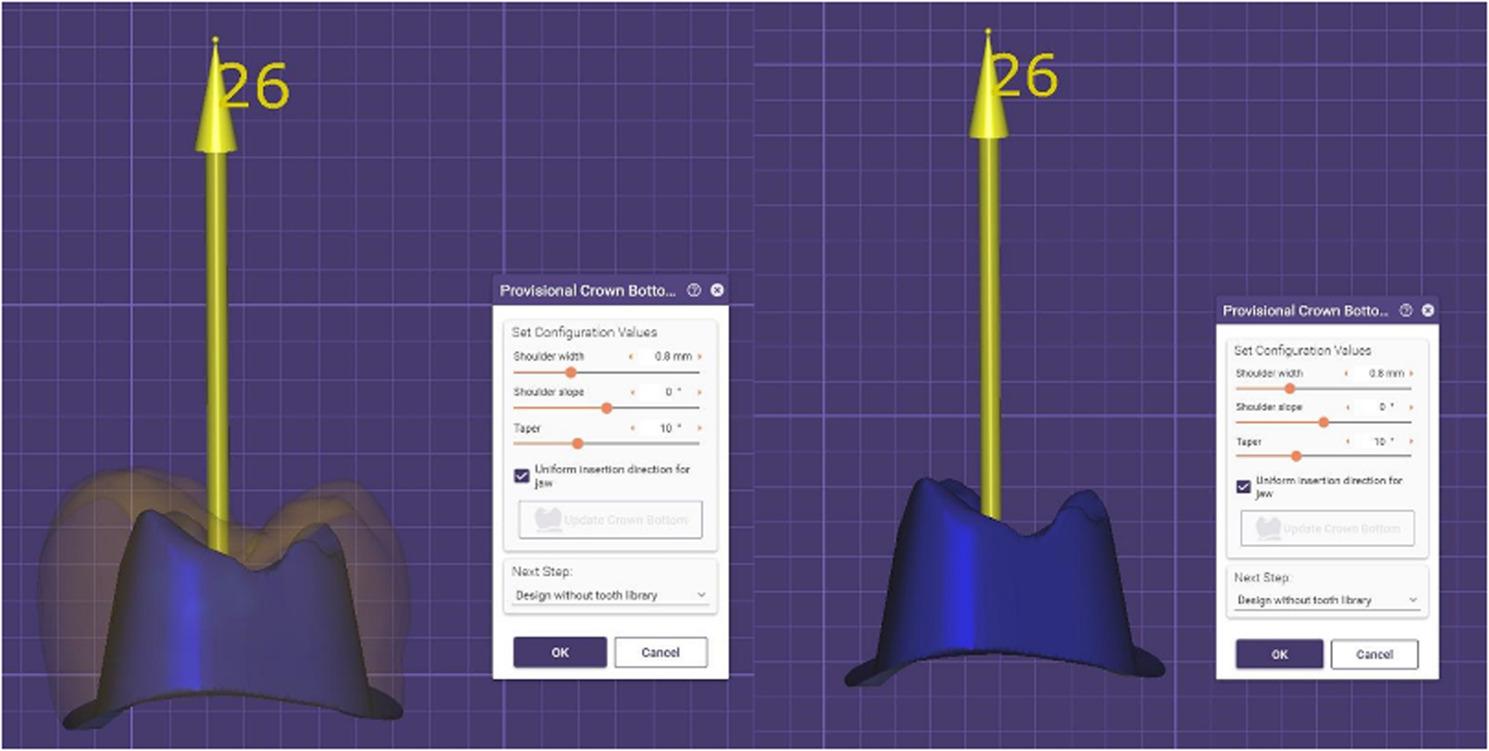
Preparation configuration designed on a Master CAD software using the “egg-shell provisional” function

**Fig. 2:**
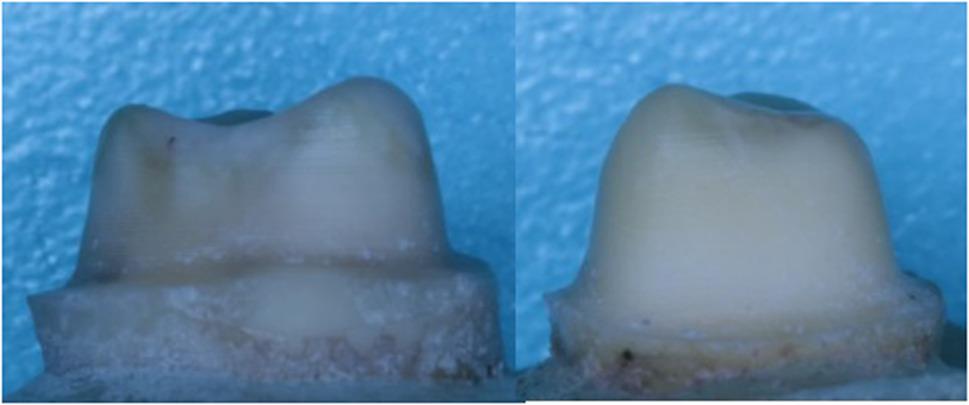
Different views of the prepared teeth

The extracted molar teeth were positioned vertically in a custom mold with a paralleling device, using acrylic resin (Acrostone, Egypt). This mold allowed exposure of only the crown and 2 mm beneath the cementoenamel junction. To ensure standardized tooth preparation across samples, a computerized numerical control milling machine (3018 CNC machine, VEVOR, United States) was utilized for preparing the teeth for full coverage crowns. After mounting, the teeth were scanned using MEDIT i700 intraoral scanner (Straumann, South Korea) used for pre-preparation morphology acquisition and the resulting scans were exported as STL files. The preparation design was created using Master CAD software (Exocad GmbH, Darmstadt, Germany) employing the “egg-shell provisional” function based on specifications for full coverage crowns (Fig. 1). The preparation included an anatomical occlusal reduction of 1.5 mm, an axial reduction of 1 mm, and an axial wall height of 4 mm. The axial preparation featured a 10-degree taper, leading to a total occlusal convergence of 20 degrees, and ended with a smooth continuous deep chamfer finish line of 0.8 mm thickness. All line and point angles were rounded to reduce stress concentration [26]. The base of the custom mold was secured to the CNC milling machine’s bed. The milling machine spindle was fitted with a TR26 round end diamond stone and aligned parallel to the tooth’s long axis in the mold. A new diamond stone was used at the start of each preparation cycle. Tooth preparation was conducted with water coolant directed at the tooth to maintain hydration and prevent overheating (Fig. 2).

Following tooth preparation and prior to the CAD/CAM fabrication of the ceramic crowns, the Research Randomizer software (https://www.randomizer.org) was utilized to randomly assign each prepared tooth to its respective testing group: Group T (Cerec Tessera) and Group E (IPS e.max CAD).

The prepared teeth were scanned with the CEREC Omnicam (Sirona Dental Systems, Bensheim, Germany), and the images were converted into 3D virtual dies. All scanning procedures were completed by a single trained operator (TS) with more than ten years of experience using the CEREC Omnicam in both clinical and in-vitro applications. Subsequently, the ceramic crowns were designed using CEREC Premium software version 4.4 (Dentsply Sirona GmbH, Germany). The restoration margin was established by marking the preparation finish line. Ten full coverage crowns were designed with parameters including a 1 mm axial wall thickness, 1.5 mm occlusal surface thickness, and an 80 μm cement space starting 1 mm above the finish lines [27]. After the design was finalized, the standard tessellation language (STL) files were transmitted to a milling machine. A ceramic block (C14) was placed in the 4-axis MC XL milling machine (Cerec-inLab MC XL, Sirona, Germany), where the milling process was fully automated. After milling, the crowns were manually removed from the block holder using a diamond cutting instrument and finished at the sprue area with a polishing kit (Ivoclar Vivadent, Schaan, Liechtenstein) using a laboratory micromotor at low speed and light pressure. Lithium disilicate crowns [IPS e.max CAD (Group E)] were milled and then glazed with FLUO Ivocolor glaze paste (Ivoclar Vivadent, Liechtenstein). The crystallization process adhered to the manufacturer’s guidelines, employing a Programat P310 furnace (Ivoclar Vivadent AG, Schaan/Liechtenstein) at 820 °C for 20 min. Advanced lithium disilicate crowns [CEREC Tessera (Group T)] were milled and subjected to a glaze matrix firing cycle, which followed the manufacturer’s recommended protocol: a standby temperature of 400 °C with a 3:30 min closing time, a heating rate of 60 °C/min up to a holding temperature of 760 °C, and a holding time of 1:30 min [28].

**Fig. 3:**
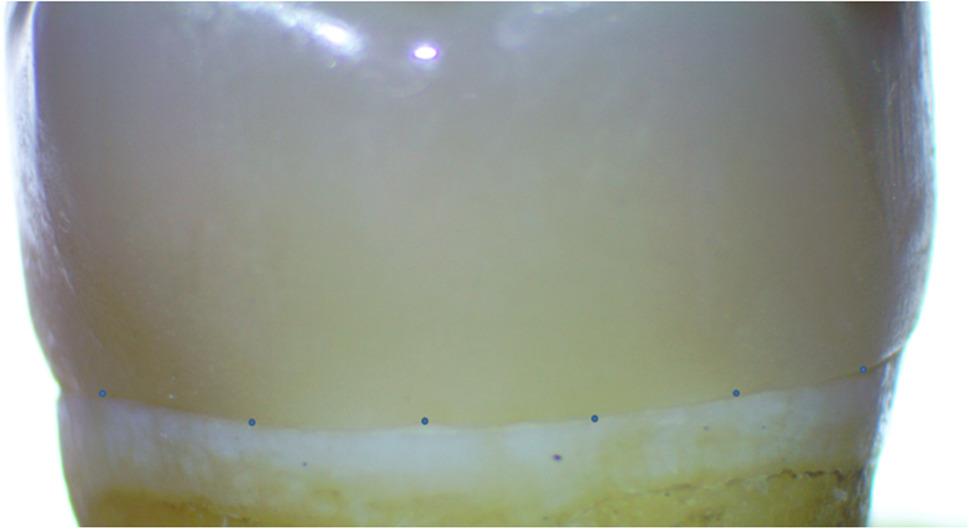
Equidistant points of measurements on stereomicroscope

The vertical marginal fit of each crown was assessed with a stereomicroscope (Euromex Microscopen BV, Arnhem, The Netherlands) equipped with a digital camera, providing 10x magnification before and after cementation. 10x magnification a sufficient field of view to capture the entire margin at each measurement point while minimizing operator error associated with focusing and image alignment. Measurements were collected at a total of 20 equidistant points for each crown (6 on the buccal, 6 on the lingual, 4 on the mesial, and 4 on the distal), and the average of these twenty points was noted for statistical analysis (Fig. 3) [26, 29–32]. Digital image analysis software (Image J 1.43U) was utilized to assess and measure the vertical marginal fit, with parameters represented in pixels and converted to microns. Standardization was achieved by referencing the measurements to a known object size (a ruler used in this study) with a scale created by the software. All measurements were performed by a single calibrated and blinded examiner. A specialized holding device was employed to secure the tested crowns to their corresponding prepared molar teeth, ensuring accurate seating during measurements prior to cementation [33].

In both the CEREC Tessera and IPS e.max CAD crown groups, the intaglio surfaces were etched with 9.5% hydrofluoric acid (BISCO’s porcelain etchant; BISCO-Schaumburg, USA) for 20 s in Group E (IPS emax CAD) samples and for 30 s in Group T (Cerec Tessera) samples. Afterward, the surfaces were rinsed and dried. A silane coupling agent (BISCO’s porcelain primer; BISCO-Schaumburg, USA) was then applied for 60 s, followed by a 5-second air spray. A self-adhesive dual-cured Totalcem resin cement (ITENA, France) was applied to the intaglio surfaces of the crowns with a mixing tip. The crowns were then carefully placed on their corresponding prepared teeth with gentle pressure.

To standardize the procedure, a 5 kg static load was applied to all samples using a cementation device for 6 min both prior to and during curing [26]. Tack curing was carried out with a light-emitting diode (LED) curing light (Elipar™, 3 M ESPE, USA) for 2–3 s from a distance of 1–2 mm, and any excess cement was carefully removed with a sharp instrument. Each surface was light-cured for 40 s to achieve full polymerization. To prevent oxygen-inhibited curing of the superficial cement layer, a glycerin gel was applied along the margins prior to the final light-curing, ensuring complete polymerization of the outermost resin layer.

After cementation, the vertical marginal fit was reevaluated using the same stereomicroscope at 10x magnification and image analysis protocol applied before cementation. To ensure measurement consistency and validity, the marginal gap was recorded at the same predefined 20 equidistant locations on each crown (6 points on the buccal, 6 on the lingual, 4 on the mesial, and 4 on the distal surfaces). The mean value of these twenty measurements was then used for statistical analysis.

### Statistical analysis of the data

The data was checked for a normal distribution by examining the data distribution and applying normality tests (Kolmogorov-Smirnov and Shapiro-Wilk tests). The results indicated that data exhibited a non-normal (non-parametric) distribution. Therefore, the data was presented using the median, range, mean and standard deviation (SD). To compare the two groups, the Mann-Whitney U test was employed. The Wilcoxon signed-rank test was employed to assess the vertical marginal fit before and after cementation. Statistical significance was established as *P* ≤ 0.05.

## Results

Table (1), indicates that the overall vertical marginal fit between the CEREC Tessera and IPS e.max CAD groups, showed no statistically significant difference, both before and after cementation.

**Table 1:** Descriptive statistics and results of Mann-Whitney U test for comparison between vertical marginal fit (µm) in the two groups

	IPS e.max CAD (*n* = 5)		CEREC Tessera (*n* = 5)		*P*- value	Effect size (d)
	Median (Range)	Mean (SD)	Median (Range)	Mean (SD)		
Before Cementation	48.5 (42.7, 75.7)	52.2 (13.7)	72 (47.8, 126.7)	76.8 (32.7)	0.175	0.951
After Cementation	18 (11.4, 45.3)	22.4 (13.6)	39.7 (22.7, 91.4)	52.3 (28.3)	0.076	1.357

Significant level at *P* ≤ 0.05

Table (2), indicates that the vertical marginal fit after cementation in both groups showed a statistically significant decrease.

**Table 2:** Descriptive statistics and results of Wilcoxon signed-rank test for comparison between vertical marginal fit (µm) before and after cementation in each group

Ceramic	Before		After		*P*-value	Effect size (d)
	Median (Range)	Mean (SD)	Median (Range)	Mean (SD		
IPS e.max CAD					0.043	0.765
	48.5 (42.7, 75.7)	52.2 (13.7)	18 (11.4, 45.3)	22.4 (10.2)		
CEREC Tessera					0.043	0.765
	72 (47.8, 126.7)	76.8 (32.7)	39.7 (22.7, 91.4)	52.3 (28.3)		

## Discussion

This in-vitro study assessed the vertical marginal fit of CAD/CAM fabricated crowns made from advanced lithium disilicate (CEREC Tessera) and conventional lithium disilicate (IPS e.max CAD). In the current study, the null hypothesis was accepted, as the results showed no statistically significant differences between the two materials in terms of vertical marginal fit.

CEREC Tessera, a recently introduced CAD/CAM block, was chosen for evaluation in this study due to its status as a unique and innovative material in the glass-matrix ceramics category. The manufacturer claims it provides up to 32% more strength than other glass ceramics and can decrease processing time by as much as 44%. Furthermore, its outstanding aesthetic qualities make it suitable for a range of dental restorations [5, 13, 34].

Various methods exist for assessing the vertical marginal fit of indirect ceramic restorations, including the direct view method using a stereomicroscope, the silicone replica technique, and the cross-sectioning technique. However, standardized guidelines for gap measurements remain lacking [20]. In this study, the direct view method was employed with a stereomicroscope set at a fixed magnification of 10x, supplemented by image analysis software. This approach is non-destructive, repeatable, and capable of providing high-precision measurements while avoiding sectioning errors associated with cross-sectional methods [35]. Evidence from previous studies indicates that direct-view assessment yields gap measurements comparable in accuracy to both the silicone replica and cross-sectional techniques, while allowing repeated evaluation at the same predefined locations [26, 36]. Vertical marginal fit was evaluated at 20 equidistant points on each crown (6 on the buccal, 6 on the lingual, 4 on the mesial, and 4 on the distal) to comprehensively assess the margin. The mean marginal fit for each crown surface was calculated from the average values of these points. This was in accordance with Aboelenein et al. [26] in 2020 and Rizk et al. [30] in 2024. This method provided adequate information regarding gap size and ensured a statistical accuracy of the results.

The vertical marginal fit of CEREC Tessera and IPS emax CAD crowns fell within the clinically acceptable range specified by McLean et al., [15] which is less than 120 μm. In this study, no statistically significant difference in the vertical marginal fit was observed between CEREC Tessera and IPS emax CAD crowns before and after cementation. This may be due to the fact that both crown materials are constructed using CAD/CAM technology, renowned for its precision and capability to produce restorations with clinically acceptable marginal fits. Other contributing factors might include the cement space settings (80 μm) and the use of self-adhesive dual-cured Totalcem resin cement for cementation. contributes to the lower viscosity of the cement, potentially enhancing sealing ability and leading to decreased vertical marginal fit values after cementation. These results are consistent with the findings of Abdelwakil et al. [10] in 2024 and Fayed et al. [3] in 2025, which also reported no statistically significant difference in vertical marginal fit values between the CEREC Tessera and IPS e.max CAD groups, both before and after cementation. This similarity may be attributed to their comparable mechanical behavior and controlled processing parameters.

IPS e.max CAD undergoes a transformation during crystallization from a glassy matrix containing 40% lithium metasilicate to a microstructure with 70% fine, interlocking lithium disilicate crystals, promoting dimensional stability during fabrication. CEREC Tessera incorporates 0.5 μm lithium disilicate and 0.2–0.3 μm virgilite platelets, with virgilite forming optimally at 800–850 °C; although differences in thermal expansion could induce microcracks, its glaze/matrix firing cycle for 4.5 to 12 min at 760 °C is designed to limit distortion [3, 4]. As marginal discrepancies mainly stem from geometric and thermal changes during machining and firing, the controlled crystallization and CAD/CAM protocols of both materials likely contributed to their similarly favorable vertical marginal adaptation.

In contrast, the findings differ from those obtained by Kojima et al. [11] in 2022 and Yamamoto et al. [12] in 2022, who reported that CEREC Tessera crowns had higher vertical marginal fit values and longer milling time (19.5 min) compared to IPS e.max CAD crowns. This variation may be attributed to differences in research methodologies and the high glass content in CEREC Tessera, which requires longer milling and could impact accuracy. Additionally, it might relate to the fact that advancements in various CAD/CAM shaping and diamond bur finishing technologies haven’t progressed as quickly as the material development. Using diamond grit burs for grinding CEREC Tessera may lead to subsurface microcracks, potentially compromising its vertical marginal fit.

Following cementation, the results of this study indicated a reduction in the mean vertical marginal fit for both the IPS e.max CAD group (Group E) and the CEREC Tessera group (Group T). This change could be due to the self-adhesive Totalcem resin cement used, which contains UDMA as a key ingredient. UDMA contributes to the lower viscosity of the cement, potentially enhancing sealing ability and leading to decreased vertical marginal fit values after cementation for both groups. Additionally, a careful cementation process using a device that applies a load of 5 kg may have contributed to the significant reduction in the final vertical marginal fit measurements of the restorations.

The findings of this study align with those of Christian et al. [21] in 2016, and Kassem et al. [22] in 2020, who reported that after cementation, the vertical marginal fit was significantly reduced when using low viscosity cements, and that the load applied during crown cementation influences the final marginal fit.

Contradictory findings were presented by Kumar et al. [23] in 2018, who observed a significant increase in vertical marginal fit following cementation. This discrepancy may be due to differences in research methodologies; their study employed self-adhesive resin cement (RelyX U100) and resin modified glass-ionomer luting cement (Relyxlut 2) with film thicknesses between 19.4 μm and 53.6 μm, without specifying any load applied during crown cementation. In contrast, this study utilized Totalcem resin cement with a film thickness of 10–15 μm and employed a cementation device with a load of 5 kg.

This study has several limitations. Vertical marginal fit was evaluated under controlled laboratory environment, which do not fully replicate intraoral variables, and the absence of aging protocols limits the ability to predict long-term behavior. The small sample size determined using an effect size from a single study reporting an unusually large effect, may overestimate statistical power and reduce generalizability of the findings. Furthermore, evaluating only vertical marginal gaps limits the comprehensiveness of the study, as other aspects of crown adaptation such as horizontal marginal gaps and internal fit warrant future investigation. Additionally, only one cement type (Totalcem) was used, which restricts comparison with other luting protocols. Therefore, the results should be interpreted with caution, and future studies with larger samples and more comprehensive assessment are recommended.

## Conclusion

Within the limitations of the current study, the following could be concluded:

1.  CEREC Tessera, a novel advanced lithium disilicate material demonstrated marginal gap values comparable to the well-established IPS e.max CAD.
2.  These findings suggest that CEREC Tessera is a clinically viable option for chairside ceramic restorations, combining accelerated fabrication with reliable adaptation, without compromising marginal integrity.

## Data Availability

The datasets used and/or analyzed during the current study are available from the corresponding author upon reasonable request.

## Abbreviations

CAD/CAM: Computer-aided design and computer-aided manufacturing technology
LDCs: Lithium disilicate-based glass ceramics
CNC: Computerized numerical control milling machine
STL: Standard tessellation language
LED: Light-emitting diode

## References

[1] Bajraktarova-Valjakova E, Korunoska-Stevkovska V, Kapusevska B, Gigovski N, Bajraktarova-Misevska C, Grozdanov A. Contemporary dental ceramic Materials, A review: chemical Composition, physical and mechanical Properties, indications for use. Open Access Maced J Med Sci. 2018;6(9):1742–55. 30338002 10.3889/oamjms.2018.378 PMC6182519

[2] Fadl AE, Zohdy A, Anwar M. Evaluation of marginal gap of CAD/CAM crowns milled from two ceramic materials. Egypt Dent J. 2018;64(3):2531–6.

[3] Fayed AK, Azer AS, AboElhassan RG. Fit accuracy and fracture resistance evaluation of advanced lithium disilicate crowns (in- vitro study). BMC Oral Health. 2025;25(1):58–69. 39799312 10.1186/s12903-024-05325-z PMC11725217

[4] Phark J, Duarte S. Microstructural considerations for novel lithium disilicate glass ceramics: A review. J Esthet Restor Dent. 2022;34(1):92–103. 34995008 10.1111/jerd.12864

[5] Marchesi G, Camurri Piloni A, Nicolin V, Turco G, Di Lenarda R. Chairside CAD/CAM materials: current trends of clinical uses. Biology. 2021;10(11):1170–81. 34827163 10.3390/biology10111170 PMC8614873

[6] Bindl A, Lüthy H, Mörmann WH. Thin-wall ceramic CAD/CAM crown copings: strength and fracture pattern. J Oral Rehabil. 2006;33(7):520–8. 16774511 10.1111/j.1365-2842.2005.01588.x

[7] Brandt S, Winter A, Lauer HC, Kollmar F, Portscher-Kim SJ, Romanos GE. IPS e.max for All-Ceramic restorations: clinical survival and success rates of Full-Coverage crowns and fixed partial dentures. Materials. 2019;12(3):462–72. 30717358 10.3390/ma12030462 PMC6384731

[8] Mounajjed R, Layton M, Azar D. The marginal fit of E.max press and E.max CAD lithium disilicate restorations: A critical review. Dent Mater J. 2016;35(6):835–44. 27546857 10.4012/dmj.2016-008

[9] Arafa Hilaly MG, Elguindy JF, Hashem RM. Assessment of vertical marginal gap and fracture resistance of Tessera anterior endocrown with two different extensions. Egypt Dent J. 2024;70(4):3601–11.

[10] Abdelwakil MM, Elguindy JF, Mohamed ME. Assessment of marginal gap and fracture resistance of emax and Tessera anterior endocrown (an invitro study). Egypt Dent J. 2024;70(4):3569–80.

[11] Kojima K, Nagaoka K, Murata Y, Yamamoto K, Akiyama S, Hokii Y, et al. Marginal adaptation of CAD/CAM milled lithium disilicate glass ceramic crowns. J Osseointegr. 2022;14(4):201–4.

[12] Yamamoto K, Murata Y, Nagaoka K, Akiyama S, Hokii Y, Fusejima F. Comparison of dimensional accuracy of lithium disilicate CAD/CAM ceramics. J Osseointegration. 2022;14(4):205–8.

[13] Freitas JS, Souza LFB, Pereira GKR, May LG. Surface properties and flexural fatigue strength of an advanced lithium disilicate. J Mech Behav Biomed Mater. 2023;147:106154. 37804677 10.1016/j.jmbbm.2023.106154

[14] Zhang Y, Vardhaman S, Rodrigues CS, Lawn BR. A critical review of dental Lithia-Based Glass–Ceramics. J Dent Res. 2023;102(3):245–53. 36645131 10.1177/00220345221142755 PMC9947811

[15] McLean JW, Von F. The Estimation of cement film thickness by an in vivo technique. Br Dent J. 1971;131(3):107–11. 5283545 10.1038/sj.bdj.4802708

[16] Heboyan AG. Marginal and internal fit of fixed prosthodontic constructions: a literature review. Int J Dent Res Rev. 2019;2:19–26.

[17] Sailer I, Makarov NA, Thoma DS, Zwahlen M, Pjetursson BE. All-ceramic or metal-ceramic tooth-supported fixed dental prostheses (FDPs)? A systematic review of the survival and complication rates. Part I: single crowns (SCs). Dent Mater. 2015;31(6):603–23. 25842099 10.1016/j.dental.2015.02.011

[18] Contrepois M, Soenen A, Bartala M, Laviole O. Marginal adaptation of ceramic crowns: A systematic review. J Prosthet Dent. 2013;110(6):447–e45410. 24120071 10.1016/j.prosdent.2013.08.003

[19] Tsirogiannis P, Reissmann DR, Heydecke G. Evaluation of the marginal fit of single-unit, complete-coverage ceramic restorations fabricated after digital and conventional impressions: A systematic review and meta-analysis. J Prosthet Dent. 2016;116(3):328–e3352. 27061627 10.1016/j.prosdent.2016.01.028

[20] Rastogi A, Kamble V. Comparative analysis of the clinical techniques used in evaluation of marginal accuracy of cast restoration using stereomicroscopy as gold standard. J Adv Prosthodont. 2011;3(2):69–75. 21814614 10.4047/jap.2011.3.2.69 PMC3141121

[21] Cristian AC, Jeanette L, Francisco MR, Guillermo P. Correlation between Microleakage and Absolute Marginal Discrepancy in Zirconia Crowns Cemented with Four Resin Luting Cements: An In Vitro Study. Int J Dent. 2016;2016(1):8084505-10. 10.1155/2016/8084505 PMC5046027 27721830

[22] Kasem AT, Sakrana AA, Ellayeh M, Özcan M. Evaluation of zirconia and zirconia-reinforced glass ceramic systems fabricated for minimal invasive preparations using a novel standardization method. J Esthet Restor Dent. 2020;32(6):560–8. 32011094 10.1111/jerd.12570

[23] Kumar TS, Shankar Y, Ravi, Modalavalasa H, Paidi S. Evaluation of marginal adaptation and microleakage of all ceramic crown systems by using two commercially available Luting agents - an invitro study. Int J Curr Res. 2018;10(8):72760–5.

[24] Kumar M, Sequeira P, Peter S, Bhat G. Sterilisation of extracted human teeth for educational use. Indian J Med Microbiol. 2005;23(4):256–8. 16327123

[25] Ahmed H, Essam E, Saleh O, El Mekkawi W. Marginal accuracy and microleakage of machinable laminate veneers. Al-Azhar Dent J Girls. 2020;7(2):239–45.

[26] Aboelenen RH, Mokhtar A, Zaghloul H. Evaluation of marginal fit and microleakage of monolithic zirconia crowns cemented by bio-active and glass ionomer cements: in vitro study. Braz Dent Sci. 2020;23(1):11–214.

[27] Hammood D, Ibraheem F. Evaluate and compare the effect of different marginal cement space parameter setting in the CAD software on the marginal and internal fitness of monolithic zirconia crowns with different types of Luting agents (A comparative in vitro study). J Res Med Dent Sci. 2020;8(1):74–80.

[28] Jakovac M, Klaser T, Radatović B, Skoko Ž, Pavić L, Žic M. Surface characterization and conductivity of two types of Lithium-Based glass ceramics after accelerating ageing. Materials. 2020;13(24):5632–42. 33321786 10.3390/ma13245632 PMC7763873

[29] Nawafleh NA, Mack F, Evans J, Mackay J, Hatamleh MM. Accuracy and reliability of methods to measure marginal adaptation of crowns and fdps: A literature review. J Prosthodont. 2013;22(5):419–28. 23289599 10.1111/jopr.12006

[30] Rizk A, El-Guindy J, Abdou A, Ashraf R, Kusumasari C, Eldin FE. Marginal adaptation and fracture resistance of virgilite-based occlusal veneers with varying thickness. BMC Oral Health. 2024;24(1):307–15. 38443910 10.1186/s12903-024-04071-6 PMC10913281

[31] Nasir MQ, Kadhim AJ. Marginal adaptation of different monolithic zirconia crowns with horizontal and vertical finish lines: A comparative in vitro study. J Dent Res Dent Clin Dent Prospects. 2023;17(4):235–41. 38584994 10.34172/joddd.2023.40589 PMC10998165

[32] Riccitiello F, Amato M, Leone R, Spagnuolo G, Sorrentino R. In vitro evaluation of the marginal fit and internal adaptation of zirconia and lithium disilicate single crowns: Micro-CT comparison between different manufacturing procedures. Open Dent J. 2018;12(1):160–72. 29854014 10.2174/1874210601812010160 PMC5952349

[33] Sidhom M, Zaghloul H, Mosleh IES, Eldwakhly E. Effect of different CAD/CAM milling and 3D printing digital fabrication techniques on the accuracy of PMMA working models and vertical marginal fit of PMMA provisional dental prosthesis: an in vitro study. Polymers. 2022;14(7):1285–104. 35406159 10.3390/polym14071285 PMC9003362

[34] Altan B, Çinar Ş, Uz BB, Özkan D. Evaluation of the marginal fit of finish line designs of novel CAD/CAM restoration materials. J Health Sci Med. 2023;6(1):116–21.

[35] Elsharkawy EE, El-Kouedi A, Shokry T. Evaluation of the vertical marginal gap of three CAD/CAM ceramic system after Cyclic loading. Al-Azhar J Dent Sci. 2023;26(3):317–23.

[36] De Kok P, Liao P, Chien EC, Morgano S. A meta-analysis of the accuracy of different measuring techniques to evaluate the marginal and internal gap of a fixed dental prosthesis: the American academy of fixed prosthodontics, research in fixed prosthodontics committee. J Prosthet Dent. 2025;134(1):42–9. 40016075 10.1016/j.prosdent.2025.01.034

